# Adaptive pulsed laser line extraction for terrain reconstruction using a dynamic vision sensor

**DOI:** 10.3389/fnins.2013.00275

**Published:** 2014-01-17

**Authors:** Christian Brandli, Thomas A. Mantel, Marco Hutter, Markus A. Höpflinger, Raphael Berner, Roland Siegwart, Tobi Delbruck

**Affiliations:** ^1^Department of Information Technology and Electrical Engineering, Institute of Neuroinformatics, ETH Zurich and University of ZurichZurich, Switzerland; ^2^Autonomous Systems Lab, Department of Mechanical and Process Engineering, ETH ZurichZurich, Switzerland

**Keywords:** neuromorphic, robotics, event-based, address-event representation (AER), dynamic vision sensor (DVS), silicon retina

## Abstract

Mobile robots need to know the terrain in which they are moving for path planning and obstacle avoidance. This paper proposes the combination of a bio-inspired, redundancy-suppressing dynamic vision sensor (DVS) with a pulsed line laser to allow fast terrain reconstruction. A stable laser stripe extraction is achieved by exploiting the sensor's ability to capture the temporal dynamics in a scene. An adaptive temporal filter for the sensor output allows a reliable reconstruction of 3D terrain surfaces. Laser stripe extractions up to pulsing frequencies of 500 Hz were achieved using a line laser of 3 mW at a distance of 45 cm using an event-based algorithm that exploits the sparseness of the sensor output. As a proof of concept, unstructured rapid prototype terrain samples have been successfully reconstructed with an accuracy of 2 mm.

## Introduction

Motion planning in mobile robots requires knowledge of the terrain structure in front of and underneath the robot; possible obstacles have to be detected and their size has to be evaluated. Especially legged robots need to know the terrain on which they are moving so that they can plan their steps accordingly. A variety of 3D scanners such as the Microsoft Kinect^©^ (Palaniappa et al., 2011) or LIDAR (Yoshitaka et al., 2006; Raibert et al., 2008) devices can be used for this task but these sensors and their computational overhead typically consume on the order of several watts of power while having a sample rate limited to tens of Hertz. Passive vision systems partially overcome these limitations but they exhibit a limited spatial resolution because their terrain reconstruction is restricted to a small set of feature points (Weiss et al., 2010).

Many of the drawbacks in existing sensor setups (active as well as passive) arise from the fact that investigating visual scenes as a stroboscopic series of (depth) frames leads to redundant data that occupies communication and processing bandwidth and limits sample rates to the frame rate. If the redundant information is already suppressed at the sensor level and the sensor asynchronously reports its output, the output can be evaluated faster and at a lower computational cost. In this paper such a vision sensor, the so called dynamic vision sensor (DVS; Lichtsteiner et al., 2008) is combined with a pulsed line laser, forming an active sensor to reconstruct the terrain in front of the system while it is moved. This terrain reconstruction is based on a series of surface profiles based on the line laser pulses. The proposed algorithm allows extracting the laser stripe from the asynchronous temporal contrast events generated by the DVS using only the event timing so that the laser can be pulsed at arbitrary frequencies from below 1 Hz up to 500 Hz. The flexibility in choosing the pulsing frequencies allows fast and detailed surface reconstructions for fast robot motions as well as saving laser power for slow motions.

### The dynamic vision sensor (DVS)

**Figure 1:**
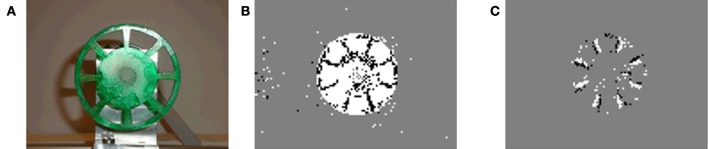
**Wheel spinning at 3000 rpm. (A)** Still image. **(B)** Events generated in 30 ms: ON events rendered white, OFF events in black. **(C)** Events generated in 200 us.

The DVS used in this setup is inspired by the functionality of the retina and senses only changes in brightness (Lichtsteiner et al., 2008). Each pixel reports a change in log-illuminance larger than a given threshold by sending out an asynchronous address-event: if it becomes brighter it generates a so called “ON event,” and if darker, it generates an “OFF event.” The asynchronously generated address-events are communicated to a synchronous processing device by a complex programmable logic device (CPLD) which also transmits the time in microseconds at which the event occurred. Each event contains the pixel horizontal and vertical address (*u,v*), its polarity (ON/OFF) and the timestamp. After the event is registered, it is written into a FIFO buffer which is transferred through a high-speed USB 2.0 interface to the processing platform. Real-time computations on the processing platform operate on the basis of so called event packets which can contain a variable number of events but are delivered at a minimum frequency of 1 kHz. This approach of sensing a visual scene has the following advantages:

1. The absence of a global exposure time lets each pixel settle to its own operating point which leads to a dynamic range of more than 120 dB.
2. Because the pixels only respond to brightness changes, the output of the sensor is non-redundant. This leads to a decrease in processor load and therefore to a reduction in power consumption of the system.
3. The asynchronous readout allows a low latency of as little as 15 us. This latency allows to close control loops very quickly as demonstrated in Delbruck and Lichtsteiner (2007); Conradt et al. (2009); Ni et al. (2012). Figure 1 shows the speed of the DVS, which is capable of resolving fast movements such as a wheel spinning at 3000 rpm.
4. Since the events are timestamped as they occur (with a temporal resolution of 1 us), the output allows a detailed analysis of the dynamics in a scene or to process its output using temporal filters.

In the following, the output of the DVS is described as a set of events and each event *Ev* carries its *u*- and *v*-address, a timestamp and its polarity as a value of +1 if it is an ON event and a −1 for OFF events [with notation adapted from Ni et al. (2012)].

(1) $$Ev(u,v,t)=\{\begin{array}{l} +1,\text{ if }\Delta \ln(I_{u,v})>\Theta_{\text{ON}}\\ -1,\text{ if }\Delta \ln(I_{u,v})<\Theta_{\text{OFF}} \end{array}$$

where Δ ln(*I*_*u*,*v*_) denotes the change in illumination at the pixel with coordinates *u,v* since the last event. Θ_ON_ and Θ_OFF_ denote the event thresholds that must be crossed to trigger an event. These thresholds can be set independently which allows balancing the number of ON and OFF events.

In addition to these visually triggered events, the DVS allows the injection of special, timestamped trigger events to the output stream by applying a pulse to a pin on the back of the sensor. These *Et* events are numbered in software so that they carry a pulse number and a timestamp:

(2) $$Et_{n}=t.$$

## Materials and methods

### Hardware setup

**Figure 2:**
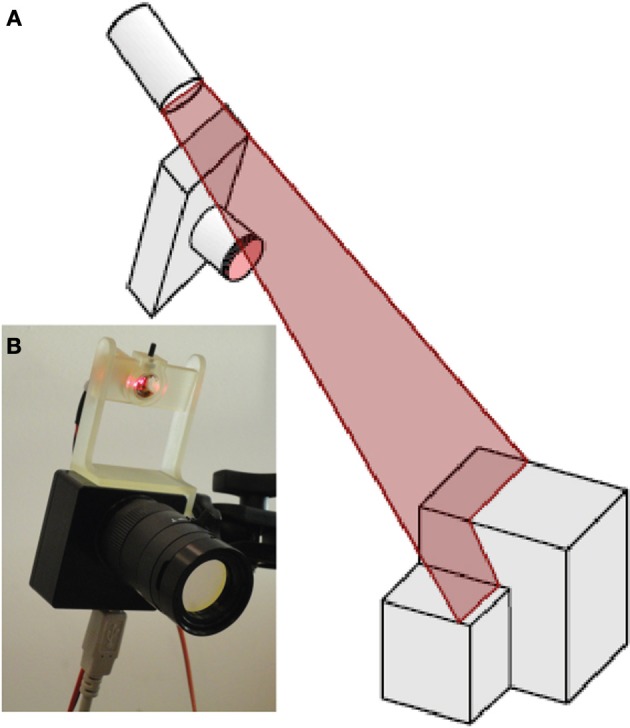
**Setup of the DVS together with the line laser. (A)** Schematic view of the setup. **(B)** Photo of the DVS128 camera with line laser: the rigid laser mount allows a constant distance and inclination angle of the laser with respect to the camera. The optical filter is mounted on the lens.

As reviewed in Forest and Salvi (2002), there are several variations of combining a line laser and a camera to build a 3D scanner. Since it is intended to apply this scanner setup on a mobile robot that already has a motion model for the purpose of navigation, a mirror free, fixed geometry setup was chosen. As shown in Figure 2, a red line laser (Laser Components GmbH LC-LML-635) with a wavelength of 635 nm and an optical power of about 3 mW was mounted at a fixed distance above the DVS. (The laser power consumption was 135 mW.) The relative angle of the laser plane and the DVS was fixed. To run the terrain reconstruction, the system is moved over the terrain while the laser is pulsed at a frequency *f*_*p*_. Each pulse of the laser initiated the acquisition of a set of events for further analysis and laser stripe extraction. The background illumination level was a brightly-lit laboratory at approximately 500 lx.

For the measurements described in the results section, the system was fixed and the terrain to scan was moved on an actuated sled on rails underneath it. This led to a straight-forward camera motion model controlled by the speed of the DC motor that pulled the sled toward the sensor system. The sled was fixed to rails which locked the system in one dimension and led to highly repeatable measurements. The DVS was equipped with a lens having a focal length of 10 mm and it was aimed at the terrain from a distance of 0.45 m. The laser module was placed at a distance of 55 mm from the sensor at an inclination angle α_*L*_ of 8° with respect to the principal axis of the DVS. The system observed the scene at an inclination angle α_*C*_ of 39°.

To enhance the signal to noise ratio, i.e., the percentage of events originating from the pulsed laser line, the sensor was equipped with an optical band pass filter (Edmund Optics NT65-167) centered at 636 nm. The filter has full width at half maximum (FWHM) of 10 nm and a transmittance of 85% in the pass band and less than 0.01% in the stop band (optical density 4.0).

To mark the laser pulses within the event stream, the event trigger pin on the back of the DVS was connected to the function generator triggering the laser.

### Calibration

**Figure 3:**
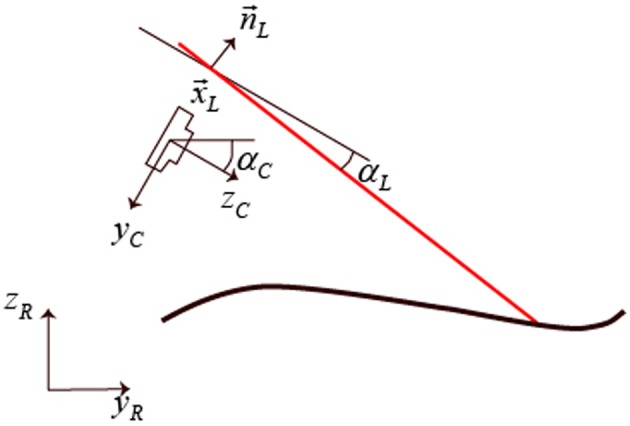
**The coordinate systems used along the scanning direction**. *y*_*R*_, *z*_*R*_ are the real world coordinates, *y*_*C*_, *z*_*C*_ the ones of the camera. *x*_*L*_ is the distance of the laser line plane perpendicular to *n*_*L*_ from the camera origin. α_*C*_ is the inclination angle of the sensor with respect to the horizontal plane and α_*L*_ the laser inclination angle with respect to the camera.

To extract the laser stripe, i.e., the pixels whose events originate from the laser line, the sensor is calibrated based on the approach described in Siegwart (2011). The model was simplified by the following assumptions:

1. For the intrinsic camera model, rectangular pixels with orthogonal coordinates *u,v* are assumed. This leads to the following transformation from pixel coordinates to camera coordinates *x*_*C*_, *y*_*C*_, *z*_*C*_:
  (3) $$u=\frac{kf_{l}}{z_{C}}x_{C}+u_{0}$$
  (4) $$v=\frac{kf_{l}}{z_{C}}y_{C}+v_{0}$$
  where *k* denotes the inverse of the pixel size, *f*_*l*_ the focal length in pixels, and *u*_0_, *v*_0_ the center pixel coordinates.
2. For the extrinsic camera model it was assumed that the rail restricts the origin of the camera *x*_*C*0_, *y*_*C*0_, *z*_*C*0_ to a planar translation (by *t*_*y*_ and *t*_*z*_) within a plane spanned by the y- and z-axis of the world reference frame *x*_*R*_, *y*_*R*_, and *z*_*R*_ as depicted in Figure 3. In the setup used for the measurement, the rotational degrees of freedom of the system were constrained so that the that the camera could only rotate (by α_*C*_) around its x-axis which leads to following transformation from camera to world coordinates:
  (5) $$\left(\begin{array}{l} x_{R}\\ y_{R}\\ z_{R} \end{array}\right)\text{​}=\text{​}\left(\begin{array}{lll} 1 & \;0 & \;0\\ 0 & \cos(\alpha_{C}+\frac{\pi }{2}) & \sin(\alpha_{C}+\frac{\pi }{2})\\ 0 & -\sin(\alpha_{C}+\frac{\pi }{2}) & \cos(\alpha_{C}+\frac{\pi }{2}) \end{array}\right)\left(\begin{array}{l} x_{C}\\ y_{C}\\ z_{C} \end{array}\right)\text{​}+\text{​}\left(\begin{array}{l} 0\\ t_{y}\\ t_{z} \end{array}\right)$$

**Figure 4:**
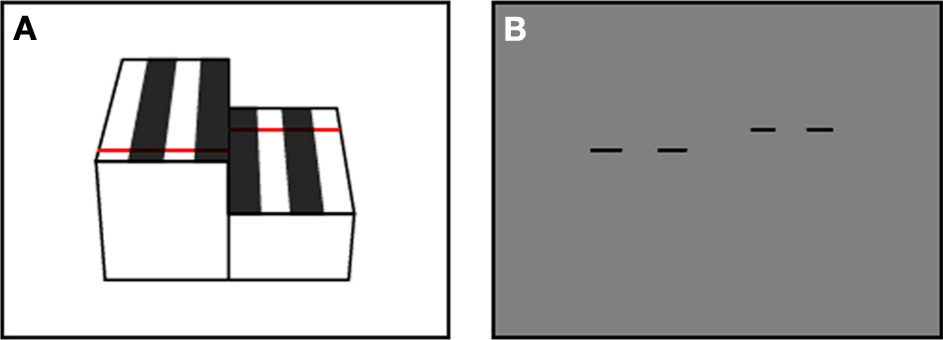
**The calibration setup**. The pulsed laser shines onto two striped blocks of different height. **(A)** Schematic view. **(B)** Schematic of the DVS output: the laser is absorbed by the black stripes and only the white stripes generate events.

The fact that the DVS does not produce any output for static scenes makes it difficult to find and align correspondences and therefore the typical checkerboard pattern could not be used for calibration. As an alternative, the laser was pulsed onto two striped blocks of different heights as depicted in Figure 4. The black stripes on the blocks absorb sufficient laser light to not excite any events in the DVS. This setup allows finding sufficient correspondence points between the real world coordinates and the pixel coordinates to solve the set of calibration equations (Equations 3–5). This procedure is done manually in Matlab but needs only to be done once.

### Laser stripe extraction

**Figure 5:**
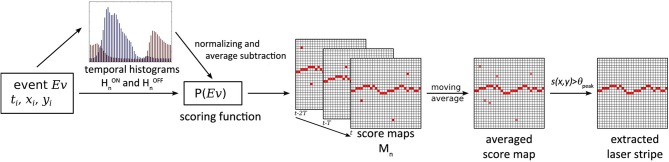
**Schematic overview of the laser stripe extraction filter**. At the arrival of each laser pulse the temporal histograms are used to adapt the scoring function P, and each event's score is calculated and mapped on the score maps. The maps are averaged and the laser stripe is extracted by selecting the maximum scoring pixel for each column, if it is above the threshold θ_peak_.

The stripe extraction method is summarized in Figure 5. Most laser stripe extraction algorithms perform a simple column-wise maximum computation to find the peak in light intensity e.g., Robinson et al. (2003); Orghidan et al. (2006). Accordingly for the DVS the simplest approach to extract the laser stripe would be to accumulate all events after a laser pulse and find the column-wise maximum in activity. This approach performs poorly due to background activity: Even with the optical filter in place, contrast edges that move relative to the sensor also induce events which corrupt the signal to noise ratio. For a more robust laser strip extraction, spatial constraints could be introduced but this would restrict the generality of the approach (Usamentiaga et al., 2010). Instead the proposed approach exploits the highly resolved temporal information of the output of the DVS.

With the help of the laser trigger events *Et*_*n*_, the event stream can be sliced into a set of time windows *W*_*n*_ each containing a set of events *S*_*n*_ where *n* denotes the *n*'th trigger event. ON and OFF events are placed into separate sets (for simplicity only the formulas for the ON events are shown):

(6) $$W_{n}=\{t:t>Et_{n}\wedge t<Et_{n+1}\}$$

(7) $$S_{n}^{\text{ON}}=\{Ev(u,v,t):t\in W_{n}\wedge Ev>0\}$$

**Figure 6:**
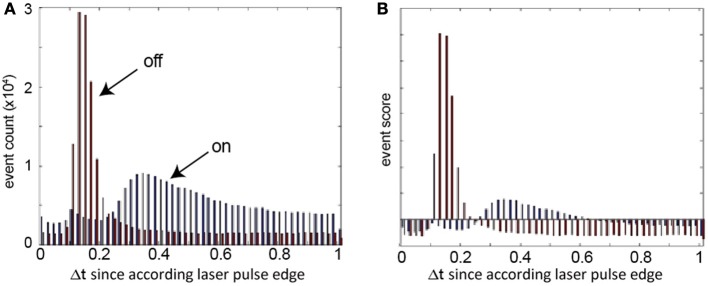
**Scoring function: examples of event histograms of the laser pulsed at 1 kHz at the relief used for the reconstruction**. **(A)** Measured histograms of ON and OFF events following laser pulse ON and OFF edges. **(B)** Resulting OFF and ON scoring functions after normalization and mean subtraction.

The timing of the events is jittered by the asynchronous communication and is also dependent on the sensor's bias settings and light conditions. Our preliminary experiments showed that it is not sufficient to only accumulate the events in a fixed time window after the pulse. Instead a stable laser stripe extraction algorithm must adaptively collect relevant events. This adaptation is achieved by using of a temporal scoring function *P* which is continually updated as illustrated in Figure 6.

The scoring function is used as follows: Each event obtains a score *s* = *P*(*Ev*) depending only on its time relative to the last trigger. From these *s* a score map *M*_*n*_ (Figure 5) is established where each pixel (*u,v*) of *M*_*n*_ contains the sum of the scores of all the events with address (*u,v*) within the set *S*_*n*_ [these subsets of *S*_*n*_ are denoted as *C*_*n*_(*u*, *v*)]. In other words, *M*_*n*_ is a 2D histogram of event scores. This score map tells us for each pixel how well-timed the events were with respect to the *n*'th trigger event, and it is computed by Equations 8–9:

(8) $$C_{n}^{\text{ON}}(u,v)=\{Ev(u^{\prime },v^{\prime },t):Ev\in S_{n}^{\text{ON}}\wedge u^{\prime }=u\wedge v^{\prime }=v\}$$

(9) $$M_{n}(u,v)=\sum_{C^{\text{ON}}(u,v)}P_{n}^{\text{ON}}(Ev)+\sum_{C^{\text{OFF}}(u,v)}P_{n}^{\text{OFF}}(Ev)$$

The scoring function *P* that assigns each event a score indicating how probable it is that it was caused by the laser pulse *Et*_*n*_ is obtained by using another histogram-based approach. The rationale behind this approach is the following: All events that are caused by the laser pulse should be temporally correlated with it while noise events should show a uniform temporal distribution. In a histogram with binned relative times the events triggered by the laser pulse should form peaks. In the proposed algorithm, the histogram *H*_*n*_ consists of *k* bins *B*_*n*_ of width *fk*. For stability, *H*_*n*_ is an average over *m* laser pulses. *H*_*n*_ is constructed by Equations 10–12:

(10) $$\begin{array}{l} D_{n}^{\text{ON}}(l)=\{Ev(u,v,t):Ev\in S_{n}^{\text{ON}}\wedge t\\ \;-Et_{n}\geq \frac{l}{fk}\wedge t-Et_{n}<\frac{l+1}{fk}\} \end{array}$$

(11) $$B_{n}^{\text{ON}}(l)=\sum_{i=n-m}^{n-1}\sum_{D_{i}^{\text{ON}}(l)}\left\|Ev\right\|$$

(12) $$H_{n}^{\text{ON}}=\left\{B_{n}^{\text{ON}}(l):l\in \left[0,k-1\right]\right\}$$

where *f* is the laser frequency, *l* is the bin index, *k* is the number of bins, *D*_*n*_(*l*) is a temporal bin of the set *S*_*n*_, *B*_*n*_(*l*) is a bin of the averaged histogram over the *m* and the histogram *H*_*n*_ is the set of all bins *B*_*n*_. It is illustrated in Figure 6A.

To obtain the scoring function *P*, the *H*^ON^_*n*_ and *H*^OFF^_*n*_ histograms are normalized by the total number *T* of events in them. To penalize bins that have a count below the average i.e., bins that are dominated by the uniformly distributed noise, the average bin count *T/k* is subtracted from each bin. An event can have a negative score. This is the case if it is more probable that it is noise than signal. *T*_*n*_ is computed from Equation 13:

(13) $$T_{n}^{\text{ON}}=\sum \text{​}\left\{B_{n}^{\text{ON}}:B_{n}^{\text{ON}}\in H_{n}^{\text{ON}}\right\}$$

The *n*'th scoring function *P_*n*_* (illustrated in Figure 6B) is computed from Equation 14:

(14) $$P_{n}^{\text{ON}}(Ev)=\frac{\sum \text{​}\left\{B_{n}^{\text{ON}}:Ev\in B_{n}^{\text{ON}}\right\}-\left(\frac{T_{n}^{\text{ON}}}{k}\right)}{T_{n}^{\text{ON}}}$$

To extract the laser stripe, the last *o* score maps are averaged and the maximum score *s*(*u,v*) and its *y* value are determined for each column. If the maximum value is above a threshold ϑ_peak_ it is considered to be a laser stripe pixel. If the neighboring pixels are also above the threshold, a weighted average is applied among them to determine the center of the laser stripe. The positions of the laser stripe are then transformed into real world coordinates using Equations 3–5 and thus mapped as surface points.

The pseudo-code shown in Algorithm 1 illustrates how the algorithm is executed: Only on the arrival of a new laser trigger event, the histograms are averaged, the score maps are averaged to an average score map and the laser stripe is extracted. Otherwise, for each DVS event only its contribution to the current score map is computed, using the current scoring function. The laser stripe extraction and computation of the scoring function operate on different time scales. While the length *o* of the moving average for the scoring function is chosen as small as possible to ensure a low latency, the number of histograms *m* to be averaged is chosen as large as possible to obtain higher stability and dampen the effect of variable background activity.

**Algorithm 1:** **Pseudo code for the laser stripe extraction**.

//iterate over all events in a packet for event:packet //the laser stripe extraction is only done at //the arrival of a new pulse if(event.isTrigger) lastTrigger = event.timestamp histogramAverage.removeOldest() histogramAverage.add(histogram) histogram.clear() //update done according to Equation (14) scoreFunction.update(histogramAverage) averageMap.removeOldest() averageMap.add(scoreMap) laserLine = averageMap.findColumPeaks() else //update of histogram deltaT = lastTrigger-event.timestamp binIndex = deltaT*k/period histogram.bin[binIndex]++ //update of score map score = scoreFunction.get(binIndex) scoreMap[event.u][event.v]+=score end if

#### Algorithm optimization

To reduce the memory consumption and the computational cost of this “frame-based” algorithm, the computations of the scoring function, the accumulation of evidence into a score map, and the search for the laser line columns were optimized to be event-based.

The average histogram changes only on a long time scale (depending on lighting conditions and sensor biasing) and this fact is exploited by only updating the averaged histogram every *m*'th pulse. The *m* histograms do not have to be memorized and each event only increases the bin count. The new score function is computed from the accumulated histogram by normalizing it only after the *m*'th pulse.

The score map computation is optimized by accumulating event scores for *o* laser pulses. Each event requires a lookup of its score and a sum into the score map. After each sum, if the new score value is higher than the previous maximum score for that column, then the new maximum score value and its location are stored for that column. This accumulation increases the latency by a factor of *o*, but is necessary in any case when the DVS events are not reliably generated by each pulse edge.

After the *o* laser pulses are accumulated, the search of the column wise maxima laser line pixels is based on the maximum values and their locations stored during accumulation. For each column, the weighted mean location of the peak is computed starting at the stored peak value and iterating over pixels up and down from the peak location until the score drops below the threshold value. This way, only a few pixels of the score map are inspected for each column.

The final step is to reset the accumulated score map and peak values to zero. This low-level memory reset is done by microprocessor logic hardware and is very fast.

Results of these optimizations are reported in Results.

### Parameter settings

Because the DVS does analog computation at the pixel level, the behavior of the sensor depends on the sensor bias settings. These settings can be used to control parameters such as the temporal contrast cutoff frequency and the threshold levels. For the experiments described in the following, the bias settings were optimized to report small as well as fast changes. These settings lead to an increase in noise events which does not affect the performance because they are filtered out successfully with the algorithm described previously. Furthermore, the biases are set to produce a clear peak in the temporal histogram of the OFF events (Figure 6). The variation in the peak form for ON and OFF events is caused by the different detection circuits for the two polarities in the pixel (Lichtsteiner et al., 2008) and different starting illumination conditions before the pulse edges.

The parameters for the algorithm are chosen heuristically: The bin size is fixed to 50 us, the scoring function average is taken over a sliding window size *m* = 1000 histograms, the stripe detection is set to average *o* = 3 probability maps, and the peak threshold for the line detection is chosen to be Θ_peak_ = 1.5.

Firstly, the performance of the stripe extraction algorithm was measured. Because the performance of the system is limited by the strength of the laser used, the capabilities of the DVS using a stronger laser were characterized to investigate the limits of the approach. Finally, a complex 3D terrain was used to assess the performance under more realistic conditions.

## Results

The laser stripe extraction results presented in the following were run in real-time as the open-source jAER-filter *FilterLaserLine* (jAER, 2007) on an Intel Core i7 975 @ 3.33 GHz Windows 7 × 64 platform using Java 1.7u45. The 3D reconstruction was run off-line in Matlab on the same platform.

Comparing the computational cost to process an event (measured in CPU time) between the frame-based and the event-based algorithm with *o* = 10 pulses showed an 1800% improvement from 900 to 50 ns per event. This improvement is a direct result of the sparse sensor output: For each laser line point update, only a few active pixels around the peak value in the score map column are considered, rather than the entire column. At the typical event rate of 500 keps observed in the terrain reconstruction example, using a laser pulse frequency of 500 Hz, a single core of this (powerful) PC is occupied 2.5% of its available processor time using the event-based algorithm. Turning off the scoring function histogram update further decreases compute time to an average of 30 ns/event, only 25 ns more than processing event packets with a “no operation” jAER filter that iterates over packets of DVS events without doing anything else.

### Extraction performance

To assess the line-detection performance of the stripe extraction algorithm, a ground truth was manually established for a scenario in which a plain block of uniform height was passed under the setup. The block was moved at about 2 cm/s to investigate the performance of the laser stripe extraction algorithm at different frequencies. In Table 1, the results of these measurements are displayed: “False positives” designates the ratio of events wrongly associated to the line over the total number of events. The performance of the algorithm drops at a frequency of 500 Hz and because the DVS should be capable of detecting temporal contrasts in the kHz regime, this was further investigated. For optimal algorithm performance, each pulse should at least excite one event per column. This is not the case for the line laser pulsed at 500 Hz because the pixel bandwidth at the laser intensity used is limited to about this frequency. Therefore, not every pulse results in a DVS event, and so the laser stripe can only be found in a few columns which leads to a degradation of the reconstruction quality.

**Table 1:** **Performance of the line extraction algorithm**.

**Frequency (Hz)**	**False positives (%)**
50	0.14
100	<0.01
200	0.03
500	5.75

*The line laser is not strong enough to perform well at frequencies above 200 Hz*.

**Figure 7:**
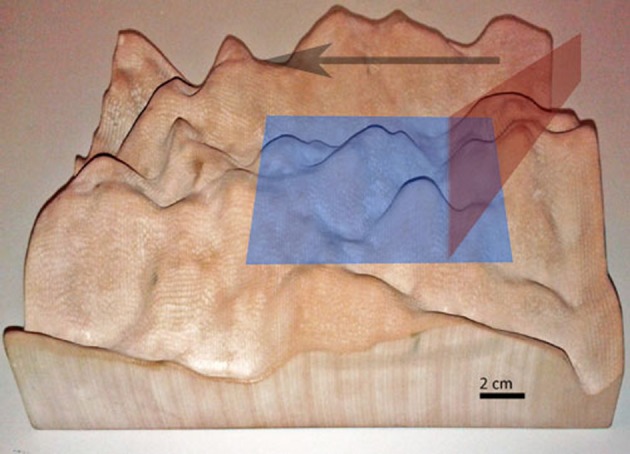
**Number of events at a pixel per laser pulse of a 4.75 mW point laser**. Although the event count drop with higher frequencies, the average does not drop below 1 event per cycle even at 2 kHz.

To explore how fast the system could go, another laser setup was used: A stronger point laser (4.75 mW, Class C) was pulsed using a mechanical shutter to avoid artifacts from the rise and fall time of the electronic driver. This point was recorded with the DVS to investigate whether it can elicit more at least one event per polarity and pulse at high frequencies. The measurements in Figure 7 show that even at frequencies exceeding 2 kHz sufficient events are triggered by the pulse. The mechanical shutter did not allow pulsing the laser faster than 2.1 kHz so the DVS might even go faster. The increase of events per pulse above 1.8 kHz is probably caused by resonances in the DVS photoreceptor circuits which facilitate the event generation. These findings indicate that a system using a sufficiently strong line laser should be capable of running at up to 2 kHz.

### Terrain reconstruction

**Figure 8:**
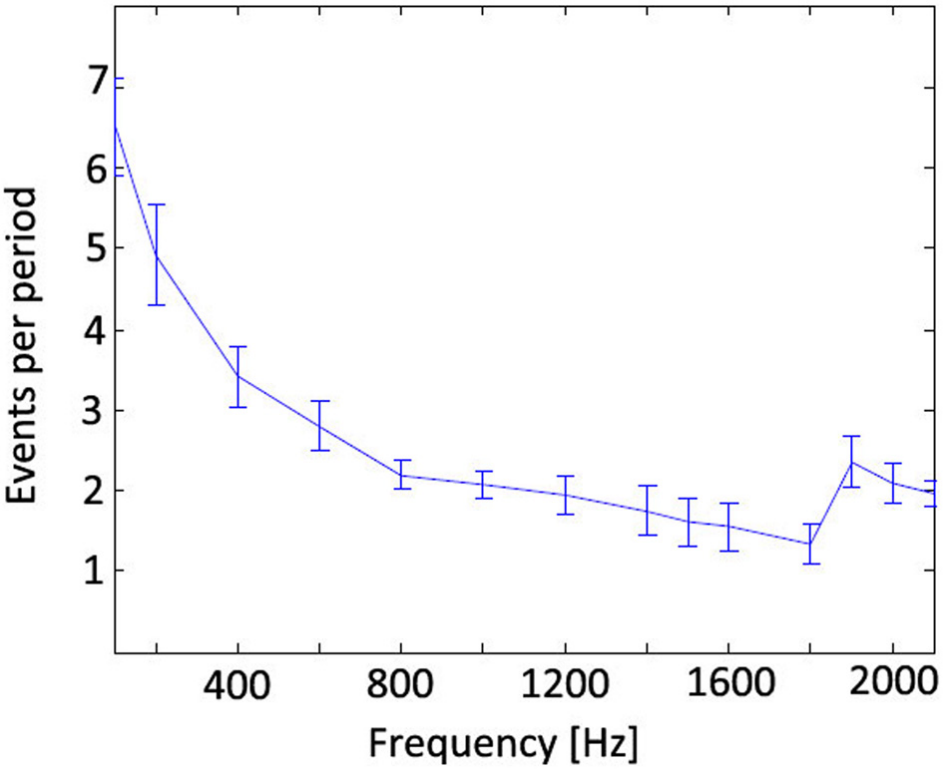
**Artificial 3D rapid prototype terrain used for proof of concept reconstruction**. Blue: area depicted in Figure 9, Red: laser line, Black: scan direction.

**Figure 9:**
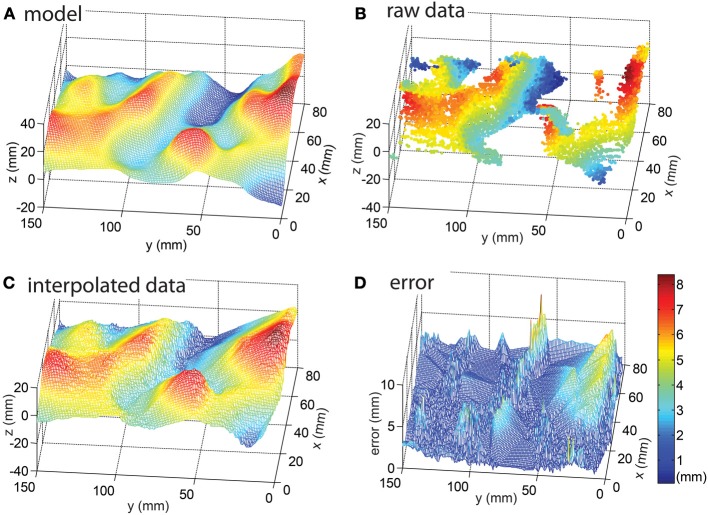
**The reconstructed surface. (A)** CAD model of the surface. **(B)** Measured data points. **(C)** Interpolated reconstruction of the surface using Matlab's *TriScatteredInterp* function. **(D)** Distance between closest reconstruction point and model aligned using ICP (Besl and McKay, 1992). This section of the reconstruction was chosen for display because in the surrounding area border effects were observed caused by the Gaussian profile of the laser line that reduced the DVS event rate to be too low to result in acceptable reconstruction.

As a proof of concept and as well for studying possible applications and shortcomings of the approach, an artificial terrain was designed with a CAD program and it was fabricated on a 3D printer (Figure 8). The sensor setup of Figure 2 was used together with the sled to capture data at a speed of 1.94 cm/s over this terrain using a laser pulse frequency of 200 Hz, translating in the *t*_*y*_ direction (Equation 5). (This slow speed was a limitation of the DC motor driving the sled.) Figure 9 shows results of these measurements: Figure 9A shows the CAD model and Figure 9B shows the raw extracted line data after transformation through Equation 5 using the calibration parameters and the measured sled speed. The blind spots where the laser did not reach the surface and the higher sampling density on front surfaces are evident. These blind spots were filled by applying the MATLAB^©^ function *TriScatteredInterp* on the sample points as shown in Figure 9C. Finally, Figure 9D shows the error between the reconstruction and model as explained in the next paragraph.

To quantify the error, the data was compared to the ground truth of the CAD model. However, the model and data lack alignment marks and therefore they were first aligned by hand using a global translation. Next, the alignment was refined using the iterative closest point algorithm (ICP; Besl and McKay, 1992), which slightly adjusted the global translation and rotation to minimize the summed absolute distance errors. Thirdly the closest 3D point of the model was determined for each point of the non-interpolated Figure 9B raw data and fourthly the distance to this model point was measured. The resulting accuracy i.e., the mean 3D distance between these two points in the 3D data is 1.7 ± 1.1 mm, i.e., the mean absolute distance between the sample and data points is 1.7 mm but the errors vary with a standard deviation of 1.1 mm. This accuracy represents ±0.25 pixel precision of measurement of the laser line given the geometry of the measurement setup. In the resampled, linearly interpolated data shown in Figure 9D, most of the error originates from the parts of the surface where the line laser is occluded by the surface, which are interpolated as flat surfaces, and in particular the bottoms of the valleys show the worst error, as could be expected.

An online movie showing the stripe extraction for the terrain reconstruction using a higher laser pulse frequency of 500 Hz is available (Adaptive filtering of DVS pulsed laser line response for terrain surface reconstruction, 2013). This video also shows various stages of the sensor output and laser line extraction.

This recording is done at a sled speed of about 1 m/s using a free-falling sled on an incline, which was not limited by the DC motor speed. In this movie it is also clear that some parts of the terrain where the laser hits the surface at a glancing angle do not generate line data. The movie also shows that background DVS activity caused by image contrast is also effectively filtered out by the algorithm although at this high frequency many pixels do not generate events on each laser pulse.

## Discussion

In this paper the first application of a DVS as a sensing device for terrain reconstruction was demonstrated. An adaptive event-based filtering algorithm for efficiently extracting the laser line position was proposed. The proposed application of DVSs in active sensor setups such as 3D scanners allows terrain reconstruction with high temporal resolution without the necessity of using a power-consuming high-speed camera and subsequent high frame rate processing or any moving parts. The event-based output of DVSs has the potential to reduce the computational load and thereby decreasing the latency and power consumption of such systems. The system benefits from the high dynamic range and the sparse output of the sensor as well as the highly resolved time information on the dynamics in a scene. With the proposed algorithm, temporal correlations between the pulsed stimulus and the recorded signal can be extracted as well as used as filtering criterion for the stripe extraction.

Further improvements to the system are necessary to realize the targeted integration to mobile robots. The Java and jAER overhead would have to be removed and the algorithm would have to be implemented on a lower level programming language (such as C) using the optimized event-based algorithm. A camera motion model and surface reconstruction would have to be integrated into the software and for portability of the system it would need to be embedded in a camera such as the eDVS (Conradt et al., 2009). Motion models could be obtained from 3D surface SLAM algorithms (Newcombe et al., 2011) and/or inertial measurement units (IMUs). The use of DVSs with a higher sensitivity (Serrano-Gotarredona and Linares-Barranco, 2013) would allow using weaker lasers to save power. Higher resolution sensors that include a static readout (Posch et al., 2011; Berner et al., 2013) would facilitate the calibration and increase the resolution. The use of a brighter line laser would allow higher laser pulsing frequencies, a wider sensing range as well as possible outdoor applications.

But despite its immature state, the proposed approach compares well to existing commercial depth sensing systems like the Microsoft Kinect^©^ and a LIDAR optimized for mobile robots such as the SOKUIKI (comparison shown in Table 2). The system has a higher maximal sampling rate than the other sensors, a much lower average latency of 5 ms at a 200 Hz pulse rate, and it is more accurate at short distances. These features are crucial for motion planning and obstacle avoidance in fast moving robots. The latency of the proposed approach is, however, dependent on the reliability of the DVS pixel responses, so there is a tradeoff between latency and noise that has not yet been fully studied, and this tradeoff will also depend on other conditions such as background lighting and surface reflectance. On the downside, the system's spatial resolution is limited by the use of the first-generation DVS128 camera and the field of view for the proposed system is narrow. But these drawbacks are not fundamental and they can easily be improved (e.g., by using newer sensors, shorter lenses and stronger lasers). The limitation that the system does not deliver depth maps but surface profiles could be overcome by projecting sparse 2D light patterns instead of a laser line. The power consumption of 500 mW for the USB camera and laser does not include the power to process the events nor to reconstruct the surface but because the sensor system power consumption is comparably lower, the data processing will probably fit into the power budget of the other two approaches when embedded into a 32-bit ARM-based microcontroller, e.g., as in Conradt et al. (2009). In summary, this paper demonstrates the applicability of DVSs combined with pulsed line lasers to provide surface profile measurement with low latency and low computational cost, but integration onto mobile platforms will require further work.

**Table 2:** **Performance comparison of the proposed approach with existing depth sensors**.

	**This work**	**Microsoft Kinect for Xbox 360**	**LIDAR (SOKUIKI)**
Spatial resolution (pixels)	128	~320 × 240^b^	680^e^
Field of view (degree)	28°	58° × 44°^b^	240°^e^
Output data	Surface profile	Depth map	Range profile
Accuracy	2 mm @ 0.45 m (0.45%)	~1.5 cm @ 3 m^c^ (0.5%)	3 cm @ 1 m^e^ (3%)
Power consumption	USB camera + laser: ~535 mW^a^	2.25–4.7 W (active)^b^	2.5 W^e^
Max sample rate (Hz)	500	30^d^	10^e^
Average latency (ms)	5^f^	45^g^–120^h^	100^e^

a *DVS:400 mW + Laser: 135 mW*.

b *Nominally 640 × 480 (Viager, 2011) but spatial pattern used reduces to ~ ±1 pixel in each direction (Andersen et al., 2012)*.

c *Khoshelham and Elberink, 2012*.

d *Kinect for Windows Sensor Components and Specifications*.

e *URG-04LX-UG01*.

f *200 Hz laser pulse rate*.

g *VGA depth map output with Core2 E6600 CPU @ 2.4 GHz (Specs about OpenNI compliant 3D sensor Carmine 1.08 | OpenNI, 2012)*.

h *Skeleton model w/1 skeleton tracked (Livingston et al., 2012)*.

### Conflict of interest statement

One of the Authors (Tobi Delbruck) is one of the research topic editors. One of the Authors (Tobi Delbruck) has a financial participation in iniLabs, the start-up which commercially distributes the DVS camera prototypes. The authors declare that the research was conducted in the absence of any commercial or financial relationships that could be construed as a potential conflict of interest.
